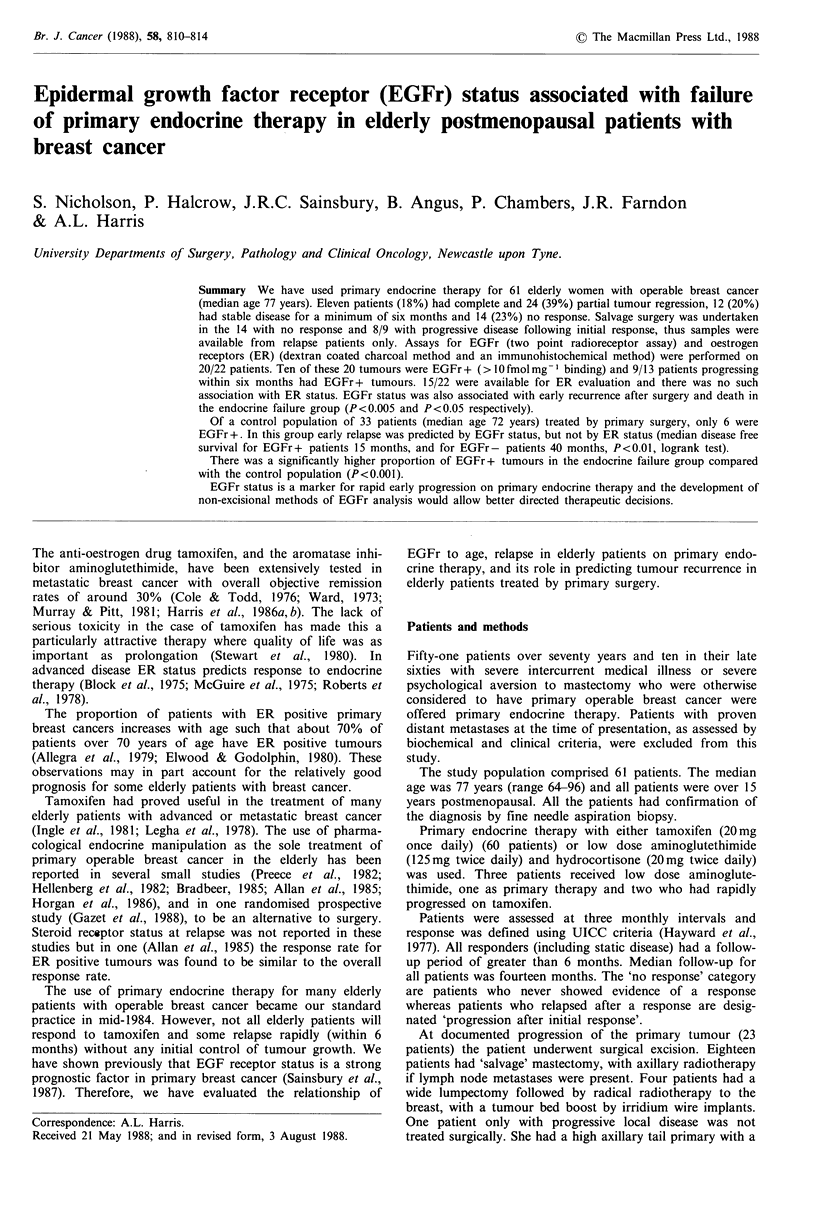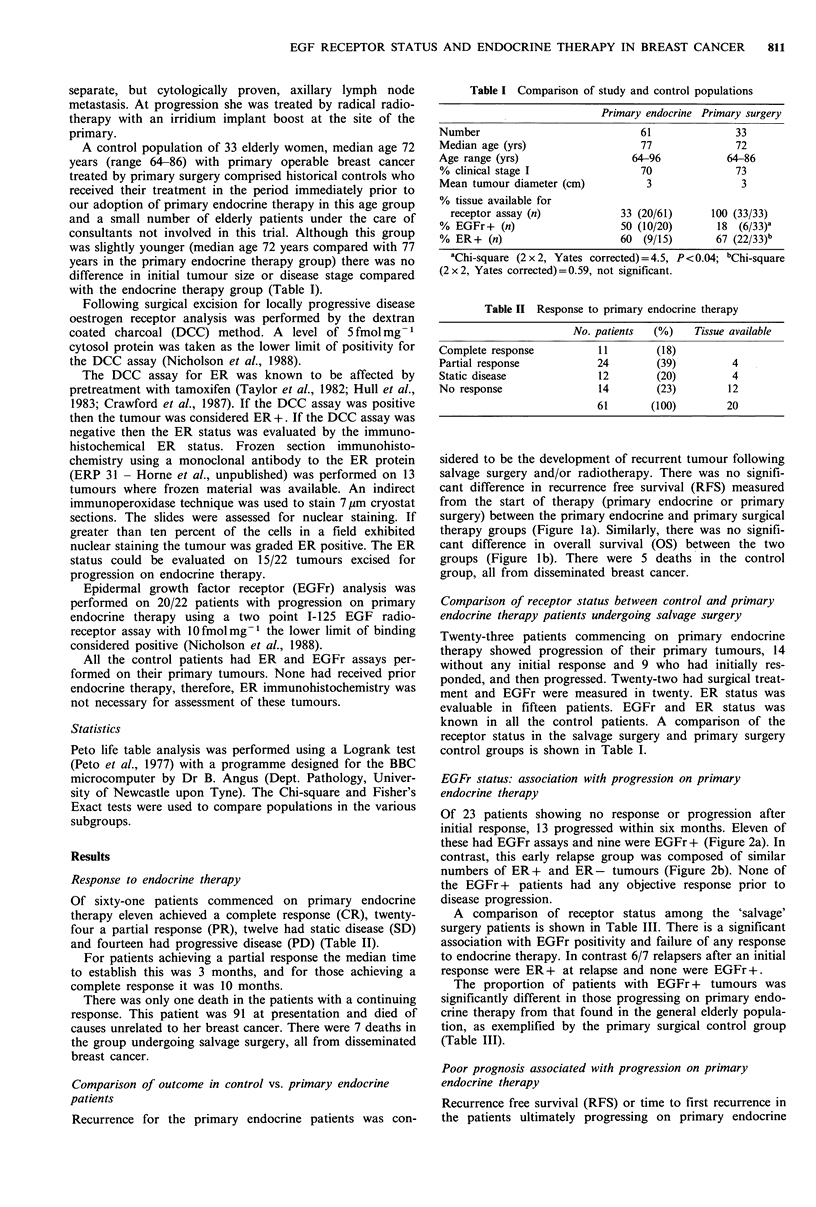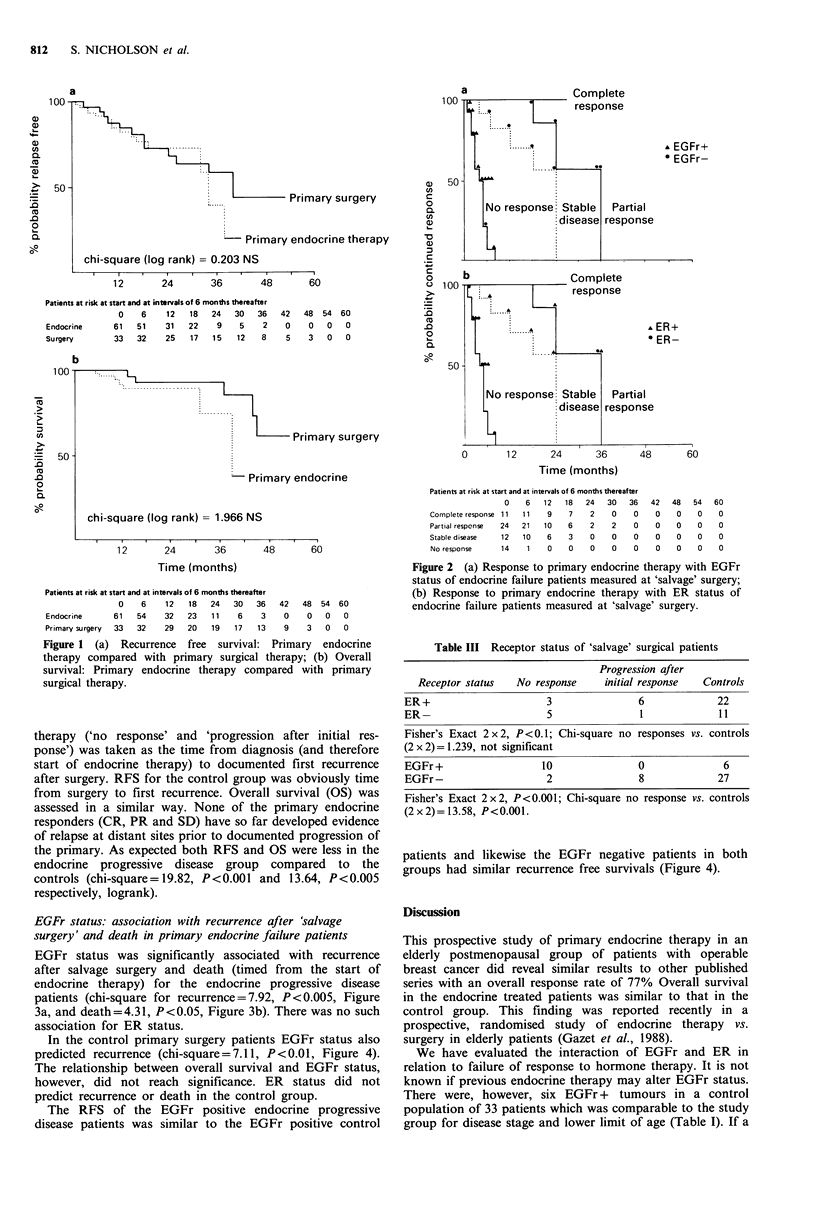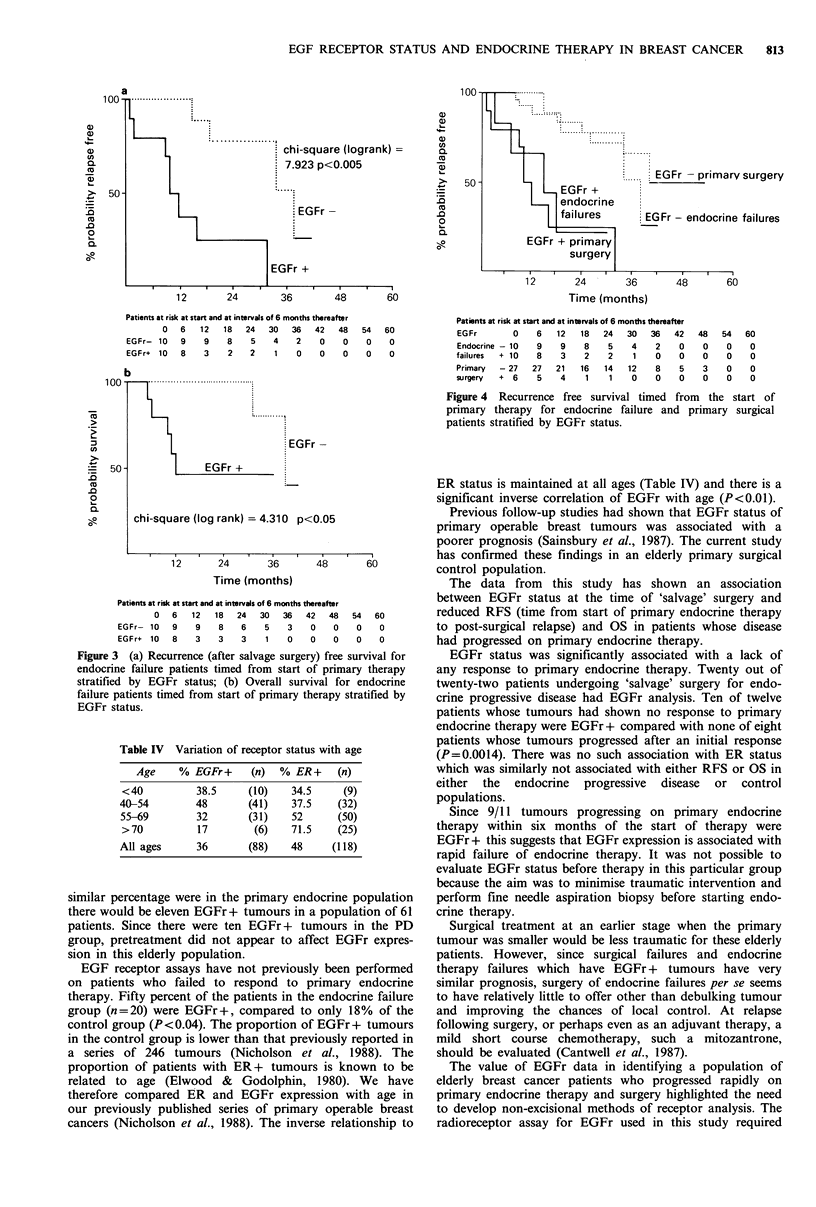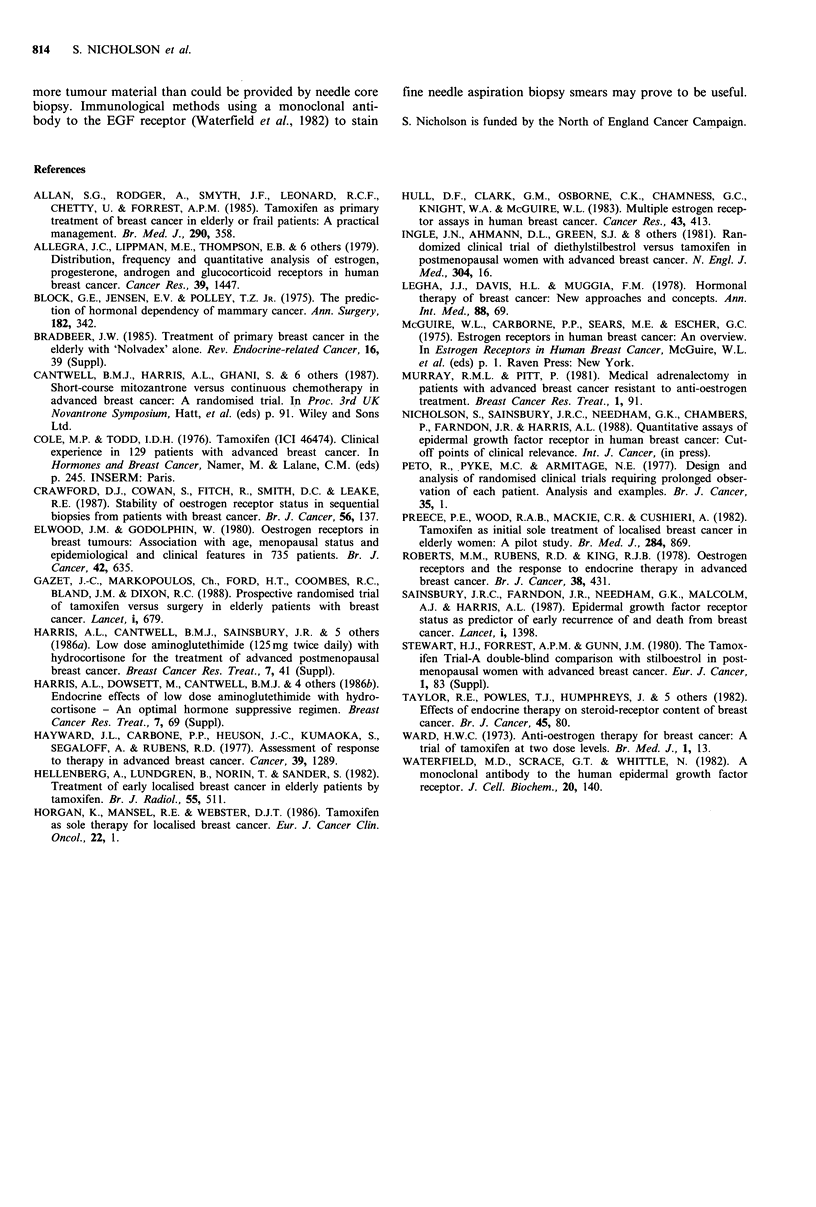# Epidermal growth factor receptor (EGFr) status associated with failure of primary endocrine therapy in elderly postmenopausal patients with breast cancer.

**DOI:** 10.1038/bjc.1988.315

**Published:** 1988-12

**Authors:** S. Nicholson, P. Halcrow, J. R. Sainsbury, B. Angus, P. Chambers, J. R. Farndon, A. L. Harris

**Affiliations:** University Department of Surgery, Newcastle upon Tyne.

## Abstract

We have used primary endocrine therapy for 61 elderly women with operable breast cancer (median age 77 years). Eleven patients (18%) had complete and 24 (39%) partial tumour regression, 12 (20%) had stable disease for a minimum of six months and 14 (23%) no response. Salvage surgery was undertaken in the 14 with no response and 8/9 with progressive disease following initial response, thus samples were available from relapse patients only. Assays for EGFr (two point radioreceptor assay) and oestrogen receptors (ER) (dextran coated charcoal method and an immunohistochemical method) were performed on 20/22 patients. Ten of these 20 tumours were EGFr+ (greater than 10 fmol mg-1 binding) and 9/13 patients progressing within six months had EGFr+ tumours. 15/22 were available for ER evaluation and there was no such association with ER status. EGFr status was also associated with early recurrence after surgery and death in the endocrine failure group (P less than 0.005 and P less than 0.05 respectively). Of a control population of 33 patients (median age 72 years) treated by primary surgery, only 6 were EGFr+. In this group early relapse was predicted by EGFr status, but not by ER status (median disease free survival for EGFr+ patients 15 months, and for EGFr- patients 40 months, P less than 0.01, logrank test). There was a significantly higher proportion of EGFr+ tumours in the endocrine failure group compared with the control population (P less than 0.001). EGFr status is a marker for rapid early progression on primary endocrine therapy and the development of non-excisional methods of EGFr analysis would allow better directed therapeutic decisions.


Br. J. Cancer (1988), 58, 810–814 © The Macmillan Press Ltd., 1988

### Epidermal growth factor receptor (EGFr) status associated with failure of primary endocrine therapy in elderly postmenopausal patients with breast cancer

S. Nicholson, P. Halcrow, J.R.C. Sainsbury, B. Angus, P. Chambers, J.R. Farndon & A.L. Harris

*University Departments of Surgery, Pathology and Clinical Oncology, Newcastle upon Tyne.*

**Summary** We have used primary endocrine therapy for 61 elderly women with operable breast cancer (median age 77 years). Eleven patients (18%) had complete and 24 (39%) partial tumour regression, 12 (20%) had stable disease for a minimum of six months and 14 (23%) no response. Salvage surgery was undertaken in the 14 with no response and 8/9 with progressive disease following initial response, thus samples were available from relapse patients only. Assays for EGFr (two point radioreceptor assay) and oestrogen receptors (ER) (dextran coated charcoal method and an immunohistochemical method) were performed on 20/22 patients. Ten of these 20 tumours were EGFr+ (>10 fmol mg$^{-1}$ binding) and 9/13 patients progressing within six months had EGFr+ tumours. 15/22 were available for ER evaluation and there was no such association with ER status. EGFr status was also associated with early recurrence after surgery and death in the endocrine failure group ($P<0.005$ and $P<0.05$ respectively).

Of a control population of 33 patients (median age 72 years) treated by primary surgery, only 6 were EGFr+. In this group early relapse was predicted by EGFr status, but not by ER status (median disease free survival for EGFr+ patients 15 months, and for EGFr− patients 40 months, $P<0.01$, logrank test).

There was a significantly higher proportion of EGFr+ tumours in the endocrine failure group compared with the control population ($P<0.001$).

EGFr status is a marker for rapid early progression on primary endocrine therapy and the development of non-excisional methods of EGFr analysis would allow better directed therapeutic decisions.

The anti-oestrogen drug tamoxifen, and the aromatase inhibitor aminoglutethimide, have been extensively tested in metastatic breast cancer with overall objective remission rates of around 30% (Cole & Todd, 1976; Ward, 1973; Murray & Pitt, 1981; Harris *et al.*, 1986*a,b*). The lack of serious toxicity in the case of tamoxifen has made this a particularly attractive therapy where quality of life was as important as prolongation (Stewart *et al.*, 1980). In advanced disease ER status predicts response to endocrine therapy (Block *et al.*, 1975; McGuire *et al.*, 1975; Roberts *et al.*, 1978).

The proportion of patients with ER positive primary breast cancers increases with age such that about 70% of patients over 70 years of age have ER positive tumours (Allegra *et al.*, 1979; Elwood & Godolphin, 1980). These observations may in part account for the relatively good prognosis for some elderly patients with breast cancer.

Tamoxifen had proved useful in the treatment of many elderly patients with advanced or metastatic breast cancer (Ingle *et al.*, 1981; Legha *et al.*, 1978). The use of pharmacological endocrine manipulation as the sole treatment of primary operable breast cancer in the elderly has been reported in several small studies (Preece *et al.*, 1982; Hellenberg *et al.*, 1982; Bradbeer, 1985; Allan *et al.*, 1985; Horgan *et al.*, 1986), and in one randomised prospective study (Gazet *et al.*, 1988), to be an alternative to surgery. Steroid receptor status at relapse was not reported in these studies but in one (Allan *et al.*, 1985) the response rate for ER positive tumours was found to be similar to the overall response rate.

The use of primary endocrine therapy for many elderly patients with operable breast cancer became our standard practice in mid-1984. However, not all elderly patients will respond to tamoxifen and some relapse rapidly (within 6 months) without any initial control of tumour growth. We have shown previously that EGF receptor status is a strong prognostic factor in primary breast cancer (Sainsbury *et al.*, 1987). Therefore, we have evaluated the relationship of EGFr to age, relapse in elderly patients on primary endocrine therapy, and its role in predicting tumour recurrence in elderly patients treated by primary surgery.

Correspondence: A.L. Harris.
Received 21 May 1988; and in revised form, 3 August 1988.

### Patients and methods

Fifty-one patients over seventy years and ten in their late sixties with severe intercurrent medical illness or severe psychological aversion to mastectomy who were otherwise considered to have primary operable breast cancer were offered primary endocrine therapy. Patients with proven distant metastases at the time of presentation, as assessed by biochemical and clinical criteria, were excluded from this study.

The study population comprised 61 patients. The median age was 77 years (range 64–96) and all patients were over 15 years postmenopausal. All the patients had confirmation of the diagnosis by fine needle aspiration biopsy.

Primary endocrine therapy with either tamoxifen (20 mg once daily) (60 patients) or low dose aminoglutethimide (125 mg twice daily) and hydrocortisone (20 mg twice daily) was used. Three patients received low dose aminoglutethimide, one as primary therapy and two who had rapidly progressed on tamoxifen.

Patients were assessed at three monthly intervals and response was defined using UICC criteria (Hayward *et al.*, 1977). All responders (including static disease) had a follow-up period of greater than 6 months. Median follow-up for all patients was fourteen months. The 'no response' category are patients who never showed evidence of a response whereas patients who relapsed after a response are designated 'progression after initial response'.

At documented progression of the primary tumour (23 patients) the patient underwent surgical excision. Eighteen patients had 'salvage' mastectomy, with axillary radiotherapy if lymph node metastases were present. Four patients had a wide lumpectomy followed by radical radiotherapy to the breast, with a tumour bed boost by irridium wire implants. One patient only with progressive local disease was not treated surgically. She had a high axillary tail primary with a separate, but cytologically proven, axillary lymph node metastasis. At progression she was treated by radical radiotherapy with an irridium implant boost at the site of the primary.

A control population of 33 elderly women, median age 72 years (range 64–86) with primary operable breast cancer treated by primary surgery comprised historical controls who received their treatment in the period immediately prior to our adoption of primary endocrine therapy in this age group and a small number of elderly patients under the care of consultants not involved in this trial. Although this group was slightly younger (median age 72 years compared with 77 years in the primary endocrine therapy group) there was no difference in initial tumour size or disease stage compared with the endocrine therapy group (Table I).

Following surgical excision for locally progressive disease oestrogen receptor analysis was performed by the dextran coated charcoal (DCC) method. A level of 5 fmol mg$^{-1}$ cytosol protein was taken as the lower limit of positivity for the DCC assay (Nicholson *et al.*, 1988).

The DCC assay for ER was known to be affected by pretreatment with tamoxifen (Taylor *et al.*, 1982; Hull *et al.*, 1983; Crawford *et al.*, 1987). If the DCC assay was positive then the tumour was considered ER+. If the DCC assay was negative then the ER status was evaluated by the immunohistochemical ER status. Frozen section immunohistochemistry using a monoclonal antibody to the ER protein (ERP 31 – Horne *et al.*, unpublished) was performed on 13 tumours where frozen material was available. An indirect immunoperoxidase technique was used to stain 7 $\mu$m cryostat sections. The slides were assessed for nuclear staining. If greater than ten percent of the cells in a field exhibited nuclear staining the tumour was graded ER positive. The ER status could be evaluated on 15/22 tumours excised for progression on endocrine therapy.

Epidermal growth factor receptor (EGFr) analysis was performed on 20/22 patients with progression on primary endocrine therapy using a two point I-125 EGF radioreceptor assay with 10 fmol mg$^{-1}$ the lower limit of binding considered positive (Nicholson *et al.*, 1988).

All the control patients had ER and EGFr assays performed on their primary tumours. None had received prior endocrine therapy, therefore, ER immunohistochemistry was not necessary for assessment of these tumours.

*Statistics*

Peto life table analysis was performed using a Logrank test (Peto *et al.*, 1977) with a programme designed for the BBC microcomputer by Dr B. Angus (Dept. Pathology, University of Newcastle upon Tyne). The Chi-square and Fisher's Exact tests were used to compare populations in the various subgroups.

### Results

*Response to endocrine therapy*

Of sixty-one patients commenced on primary endocrine therapy eleven achieved a complete response (CR), twenty-four a partial response (PR), twelve had static disease (SD) and fourteen had progressive disease (PD) (Table II).

For patients achieving a partial response the median time to establish this was 3 months, and for those achieving a complete response it was 10 months.

There was only one death in the patients with a continuing response. This patient was 91 at presentation and died of causes unrelated to her breast cancer. There were 7 deaths in the group undergoing salvage surgery, all from disseminated breast cancer.

*Comparison of outcome in control* vs. *primary endocrine patients*

Recurrence for the primary endocrine patients was considered to be the development of recurrent tumour following salvage surgery and/or radiotherapy. There was no significant difference in recurrence free survival (RFS) measured from the start of therapy (primary endocrine or primary surgery) between the primary endocrine and primary surgical therapy groups (Figure 1a). Similarly, there was no significant difference in overall survival (OS) between the two groups (Figure 1b). There were 5 deaths in the control group, all from disseminated breast cancer.

**Table I** Comparison of study and control populations

| | *Primary endocrine* | *Primary surgery* |
|---|---|---|
| Number | 61 | 33 |
| Median age (yrs) | 77 | 72 |
| Age range (yrs) | 64–96 | 64–86 |
| % clinical stage I | 70 | 73 |
| Mean tumour diameter (cm) | 3 | 3 |
| % tissue available for receptor assay (*n*) | 33 (20/61) | 100 (33/33) |
| % EGFr+ (*n*) | 50 (10/20) | 18 (6/33)[a] |
| % ER+ (*n*) | 60 (9/15) | 67 (22/33)[b] |

[a]Chi-square (2×2, Yates corrected) = 4.5, $P<0.04$; [b]Chi-square (2×2, Yates corrected) = 0.59, not significant.

**Table II** Response to primary endocrine therapy

| | *No. patients* | (%) | *Tissue available* |
|---|---|---|---|
| Complete response | 11 | (18) | |
| Partial response | 24 | (39) | 4 |
| Static disease | 12 | (20) | 4 |
| No response | 14 | (23) | 12 |
| | 61 | (100) | 20 |

*Comparison of receptor status between control and primary endocrine therapy patients undergoing salvage surgery*

Twenty-three patients commencing on primary endocrine therapy showed progression of their primary tumours, 14 without any initial response and 9 who had initially responded, and then progressed. Twenty-two had surgical treatment and EGFr were measured in twenty. ER status was evaluable in fifteen patients. EGFr and ER status was known in all the control patients. A comparison of the receptor status in the salvage surgery and primary surgery control groups is shown in Table I.

*EGFr status: association with progression on primary endocrine therapy*

Of 23 patients showing no response or progression after initial response, 13 progressed within six months. Eleven of these had EGFr assays and nine were EGFr+ (Figure 2a). In contrast, this early relapse group was composed of similar numbers of ER+ and ER− tumours (Figure 2b). None of the EGFr+ patients had any objective response prior to disease progression.

A comparison of receptor status among the 'salvage' surgery patients is shown in Table III. There is a significant association with EGFr positivity and failure of any response to endocrine therapy. In contrast 6/7 relapsers after an initial response were ER+ at relapse and none were EGFr+.

The proportion of patients with EGFr+ tumours was significantly different in those progressing on primary endocrine therapy from that found in the general elderly population, as exemplified by the primary surgical control group (Table III).

*Poor prognosis associated with progression on primary endocrine therapy*

Recurrence free survival (RFS) or time to first recurrence in the patients ultimately progressing on primary endocrine

Patients at risk at start and at intervals of 6 months thereafter

| | 0 | 6 | 12 | 18 | 24 | 30 | 36 | 42 | 48 | 54 | 60 |
|---|---|---|---|---|---|---|---|---|---|---|---|
| Endocrine | 61 | 51 | 31 | 22 | 9 | 5 | 2 | 0 | 0 | 0 | 0 |
| Surgery | 33 | 32 | 25 | 17 | 15 | 12 | 8 | 5 | 3 | 0 | 0 |

Patients at risk at start and at intervals of 6 months thereafter

| | 0 | 6 | 12 | 18 | 24 | 30 | 36 | 42 | 48 | 54 | 60 |
|---|---|---|---|---|---|---|---|---|---|---|---|
| Endocrine | 61 | 54 | 32 | 23 | 11 | 6 | 3 | 0 | 0 | 0 | 0 |
| Primary surgery | 33 | 32 | 29 | 20 | 19 | 17 | 13 | 9 | 3 | 0 | 0 |

**Figure 1** (a) Recurrence free survival: Primary endocrine therapy compared with primary surgical therapy; (b) Overall survival: Primary endocrine therapy compared with primary surgical therapy.

therapy ('no response' and 'progression after initial response') was taken as the time from diagnosis (and therefore start of endocrine therapy) to documented first recurrence after surgery. RFS for the control group was obviously time from surgery to first recurrence. Overall survival (OS) was assessed in a similar way. None of the primary endocrine responders (CR, PR and SD) have so far developed evidence of relapse at distant sites prior to documented progression of the primary. As expected both RFS and OS were less in the endocrine progressive disease group compared to the controls (chi-square = 19.82, $P<0.001$ and 13.64, $P<0.005$ respectively, logrank).

*EGFr status: association with recurrence after 'salvage surgery' and death in primary endocrine failure patients*

EGFr status was significantly associated with recurrence after salvage surgery and death (timed from the start of endocrine therapy) for the endocrine progressive disease patients (chi-square for recurrence = 7.92, $P<0.005$, Figure 3a, and death = 4.31, $P<0.05$, Figure 3b). There was no such association for ER status.

In the control primary surgery patients EGFr status also predicted recurrence (chi-square = 7.11, $P<0.01$, Figure 4). The relationship between overall survival and EGFr status, however, did not reach significance. ER status did not predict recurrence or death in the control group.

The RFS of the EGFr positive endocrine progressive disease patients was similar to the EGFr positive control

Patients at risk at start and at intervals of 6 months thereafter

| | 0 | 6 | 12 | 18 | 24 | 30 | 36 | 42 | 48 | 54 | 60 |
|---|---|---|---|---|---|---|---|---|---|---|---|
| Complete response | 11 | 11 | 9 | 7 | 2 | 0 | 0 | 0 | 0 | 0 | 0 |
| Partial response | 24 | 21 | 10 | 6 | 2 | 2 | 0 | 0 | 0 | 0 | 0 |
| Stable disease | 12 | 10 | 6 | 3 | 0 | 0 | 0 | 0 | 0 | 0 | 0 |
| No response | 14 | 1 | 0 | 0 | 0 | 0 | 0 | 0 | 0 | 0 | 0 |

**Figure 2** (a) Response to primary endocrine therapy with EGFr status of endocrine failure patients measured at 'salvage' surgery; (b) Response to primary endocrine therapy with ER status of endocrine failure patients measured at 'salvage' surgery.

**Table III** Receptor status of 'salvage' surgical patients

| *Receptor status* | *No response* | *Progression after initial response* | *Controls* |
|---|---|---|---|
| ER+ | 3 | 6 | 22 |
| ER− | 5 | 1 | 11 |
| Fisher's Exact 2 × 2, $P<0.1$; Chi-square no responses *vs.* controls (2 × 2) = 1.239, not significant | | | |
| EGFr+ | 10 | 0 | 6 |
| EGFr− | 2 | 8 | 27 |
| Fisher's Exact 2 × 2, $P<0.001$; Chi-square no response *vs.* controls (2 × 2) = 13.58, $P<0.001$. | | | |

patients and likewise the EGFr negative patients in both groups had similar recurrence free survivals (Figure 4).

### Discussion

This prospective study of primary endocrine therapy in an elderly postmenopausal group of patients with operable breast cancer did reveal similar results to other published series with an overall response rate of 77% Overall survival in the endocrine treated patients was similar to that in the control group. This finding was reported recently in a prospective, randomised study of endocrine therapy *vs.* surgery in elderly patients (Gazet *et al.*, 1988).

We have evaluated the interaction of EGFr and ER in relation to failure of response to hormone therapy. It is not known if previous endocrine therapy may alter EGFr status. There were, however, six EGFr+ tumours in a control population of 33 patients which was comparable to the study group for disease stage and lower limit of age (Table I). If a

Patients at risk at start and at intervals of 6 months thereafter

| | 0 | 6 | 12 | 18 | 24 | 30 | 36 | 42 | 48 | 54 | 60 |
|---|---|---|---|---|---|---|---|---|---|---|---|
| EGFr– | 10 | 9 | 9 | 8 | 5 | 4 | 2 | 0 | 0 | 0 | 0 |
| EGFr+ | 10 | 8 | 3 | 2 | 2 | 1 | 0 | 0 | 0 | 0 | 0 |

Patients at risk at start and at intervals of 6 months thereafter

| | 0 | 6 | 12 | 18 | 24 | 30 | 36 | 42 | 48 | 54 | 60 |
|---|---|---|---|---|---|---|---|---|---|---|---|
| EGFr– | 10 | 9 | 9 | 8 | 6 | 5 | 3 | 0 | 0 | 0 | 0 |
| EGFr+ | 10 | 8 | 3 | 3 | 3 | 1 | 0 | 0 | 0 | 0 | 0 |

**Figure 3** (a) Recurrence (after salvage surgery) free survival for endocrine failure patients timed from start of primary therapy stratified by EGFr status; (b) Overall survival for endocrine failure patients timed from start of primary therapy stratified by EGFr status.

Patients at risk at start and at intervals of 6 months thereafter

| EGFr | | 0 | 6 | 12 | 18 | 24 | 30 | 36 | 42 | 48 | 54 | 60 |
|---|---|---|---|---|---|---|---|---|---|---|---|---|
| Endocrine | – | 10 | 9 | 9 | 8 | 5 | 4 | 2 | 0 | 0 | 0 | 0 |
| failures | + | 10 | 8 | 3 | 2 | 2 | 1 | 0 | 0 | 0 | 0 | 0 |
| Primary | – | 27 | 27 | 21 | 16 | 14 | 12 | 8 | 5 | 3 | 0 | 0 |
| surgery | + | 6 | 5 | 4 | 1 | 1 | 0 | 0 | 0 | 0 | 0 | 0 |

**Figure 4** Recurrence free survival timed from the start of primary therapy for endocrine failure and primary surgical patients stratified by EGFr status.

**Table IV** Variation of receptor status with age

| *Age* | % *EGFr*+ | (*n*) | % *ER*+ | (*n*) |
|---|---|---|---|---|
| <40 | 38.5 | (10) | 34.5 | (9) |
| 40–54 | 48 | (41) | 37.5 | (32) |
| 55–69 | 32 | (31) | 52 | (50) |
| >70 | 17 | (6) | 71.5 | (25) |
| All ages | 36 | (88) | 48 | (118) |

similar percentage were in the primary endocrine population there would be eleven EGFr+ tumours in a population of 61 patients. Since there were ten EGFr+ tumours in the PD group, pretreatment did not appear to affect EGFr expression in this elderly population.

EGF receptor assays have not previously been performed on patients who failed to respond to primary endocrine therapy. Fifty percent of the patients in the endocrine failure group ($n=20$) were EGFr+, compared to only 18% of the control group ($P<0.04$). The proportion of EGFr+ tumours in the control group is lower than that previously reported in a series of 246 tumours (Nicholson *et al.*, 1988). The proportion of patients with ER+ tumours is known to be related to age (Elwood & Godolphin, 1980). We have therefore compared ER and EGFr expression with age in our previously published series of primary operable breast cancers (Nicholson *et al.*, 1988). The inverse relationship to ER status is maintained at all ages (Table IV) and there is a significant inverse correlation of EGFr with age ($P<0.01$).

Previous follow-up studies had shown that EGFr status of primary operable breast tumours was associated with a poorer prognosis (Sainsbury *et al.*, 1987). The current study has confirmed these findings in an elderly primary surgical control population.

The data from this study has shown an association between EGFr status at the time of 'salvage' surgery and reduced RFS (time from start of primary endocrine therapy to post-surgical relapse) and OS in patients whose disease had progressed on primary endocrine therapy.

EGFr status was significantly associated with a lack of any response to primary endocrine therapy. Twenty out of twenty-two patients undergoing 'salvage' surgery for endocrine progressive disease had EGFr analysis. Ten of twelve patients whose tumours had shown no response to primary endocrine therapy were EGFr+ compared with none of eight patients whose tumours progressed after an initial response ($P=0.0014$). There was no such association with ER status which was similarly not associated with either RFS or OS in either the endocrine progressive disease or control populations.

Since 9/11 tumours progressing on primary endocrine therapy within six months of the start of therapy were EGFr+ this suggests that EGFr expression is associated with rapid failure of endocrine therapy. It was not possible to evaluate EGFr status before therapy in this particular group because the aim was to minimise traumatic intervention and perform fine needle aspiration biopsy before starting endocrine therapy.

Surgical treatment at an earlier stage when the primary tumour was smaller would be less traumatic for these elderly patients. However, since surgical failures and endocrine therapy failures which have EGFr+ tumours have very similar prognosis, surgery of endocrine failures *per se* seems to have relatively little to offer other than debulking tumour and improving the chances of local control. At relapse following surgery, or perhaps even as an adjuvant therapy, a mild short course chemotherapy, such a mitozantrone, should be evaluated (Cantwell *et al.*, 1987).

The value of EGFr data in identifying a population of elderly breast cancer patients who progressed rapidly on primary endocrine therapy and surgery highlighted the need to develop non-excisional methods of receptor analysis. The radioreceptor assay for EGFr used in this study required more tumour material than could be provided by needle core biopsy. Immunological methods using a monoclonal antibody to the EGF receptor (Waterfield et al., 1982) to stain fine needle aspiration biopsy smears may prove to be useful.

S. Nicholson is funded by the North of England Cancer Campaign.

### References

ALLAN, S.G., RODGER, A., SMYTH, J.F., LEONARD, R.C.F., CHETTY, U. & FORREST, A.P.M. (1985). Tamoxifen as primary treatment of breast cancer in elderly or frail patients: A practical management. *Br. Med. J.*, **290**, 358.

ALLEGRA, J.C., LIPPMAN, M.E., THOMPSON, E.B. & 6 others (1979). Distribution, frequency and quantitative analysis of estrogen, progesterone, androgen and glucocorticoid receptors in human breast cancer. *Cancer Res.*, **39**, 1447.

BLOCK, G.E., JENSEN, E.V. & POLLEY, T.Z. JR. (1975). The prediction of hormonal dependency of mammary cancer. *Ann. Surgery*, **182**, 342.

BRADBEER, J.W. (1985). Treatment of primary breast cancer in the elderly with 'Nolvadex' alone. *Rev. Endocrine-related Cancer*, **16**, 39 (Suppl).

CANTWELL, B.M.J., HARRIS, A.L., GHANI, S. & 6 others (1987). Short-course mitozantrone versus continuous chemotherapy in advanced breast cancer: A randomised trial. In *Proc. 3rd UK Novantrone Symposium*, Hatt, et al. (eds) p. 91. Wiley and Sons Ltd.

COLE, M.P. & TODD, I.D.H. (1976). Tamoxifen (ICI 46474). Clinical experience in 129 patients with advanced breast cancer. In *Hormones and Breast Cancer*, Namer, M. & Lalane, C.M. (eds) p. 245. INSERM: Paris.

CRAWFORD, D.J., COWAN, S., FITCH, R., SMITH, D.C. & LEAKE, R.E. (1987). Stability of oestrogen receptor status in sequential biopsies from patients with breast cancer. *Br. J. Cancer*, **56**, 137.

ELWOOD, J.M. & GODOLPHIN, W. (1980). Oestrogen receptors in breast tumours: Association with age, menopausal status and epidemiological and clinical features in 735 patients. *Br. J. Cancer*, **42**, 635.

GAZET, J.-C., MARKOPOULOS, Ch., FORD, H.T., COOMBES, R.C., BLAND, J.M. & DIXON, R.C. (1988). Prospective randomised trial of tamoxifen versus surgery in elderly patients with breast cancer. *Lancet*, **i**, 679.

HARRIS, A.L., CANTWELL, B.M.J., SAINSBURY, J.R. & 5 others (1986*a*). Low dose aminoglutethimide (125mg twice daily) with hydrocortisone for the treatment of advanced postmenopausal breast cancer. *Breast Cancer Res. Treat.*, **7**, 41 (Suppl).

HARRIS, A.L., DOWSETT, M., CANTWELL, B.M.J. & 4 others (1986*b*). Endocrine effects of low dose aminoglutethimide with hydrocortisone – An optimal hormone suppressive regimen. *Breast Cancer Res. Treat.*, **7**, 69 (Suppl).

HAYWARD, J.L., CARBONE, P.P., HEUSON, J.-C., KUMAOKA, S., SEGALOFF, A. & RUBENS, R.D. (1977). Assessment of response to therapy in advanced breast cancer. *Cancer*, **39**, 1289.

HELLENBERG, A., LUNDGREN, B., NORIN, T. & SANDER, S. (1982). Treatment of early localised breast cancer in elderly patients by tamoxifen. *Br. J. Radiol.*, **55**, 511.

HORGAN, K., MANSEL, R.E. & WEBSTER, D.J.T. (1986). Tamoxifen as sole therapy for localised breast cancer. *Eur. J. Cancer Clin. Oncol.*, **22**, 1.

HULL, D.F., CLARK, G.M., OSBORNE, C.K., CHAMNESS, G.C., KNIGHT, W.A. & MCGUIRE, W.L. (1983). Multiple estrogen receptor assays in human breast cancer. *Cancer Res.*, **43**, 413.

INGLE, J.N., AHMANN, D.L., GREEN, S.J. & 8 others (1981). Randomized clinical trial of diethylstilbestrol versus tamoxifen in postmenopausal women with advanced breast cancer. *N. Engl. J. Med.*, **304**, 16.

LEGHA, J.J., DAVIS, H.L. & MUGGIA, F.M. (1978). Hormonal therapy of breast cancer: New approaches and concepts. *Ann. Int. Med.*, **88**, 69.

MCGUIRE, W.L., CARBORNE, P.P., SEARS, M.E. & ESCHER, G.C. (1975). Estrogen receptors in human breast cancer: An overview. In *Estrogen Receptors in Human Breast Cancer*, McGuire, W.L. *et al.* (eds) p. 1. Raven Press: New York.

MURRAY, R.M.L. & PITT, P. (1981). Medical adrenalectomy in patients with advanced breast cancer resistant to anti-oestrogen treatment. *Breast Cancer Res. Treat.*, **1**, 91.

NICHOLSON, S., SAINSBURY, J.R.C., NEEDHAM, G.K., CHAMBERS, P., FARNDON, J.R. & HARRIS, A.L. (1988). Quantitative assays of epidermal growth factor receptor in human breast cancer: Cut-off points of clinical relevance. *Int. J. Cancer*, (in press).

PETO, R., PYKE, M.C. & ARMITAGE, N.E. (1977). Design and analysis of randomised clinical trials requiring prolonged observation of each patient. Analysis and examples. *Br. J. Cancer*, **35**, 1.

PREECE, P.E., WOOD, R.A.B., MACKIE, C.R. & CUSHIERI, A. (1982). Tamoxifen as initial sole treatment of localised breast cancer in elderly women: A pilot study. *Br. Med. J.*, **284**, 869.

ROBERTS, M.M., RUBENS, R.D. & KING, R.J.B. (1978). Oestrogen receptors and the response to endocrine therapy in advanced breast cancer. *Br. J. Cancer*, **38**, 431.

SAINSBURY, J.R.C., FARNDON, J.R., NEEDHAM, G.K., MALCOLM, A.J. & HARRIS, A.L. (1987). Epidermal growth factor receptor status as predictor of early recurrence of and death from breast cancer. *Lancet*, **i**, 1398.

STEWART, H.J., FORREST, A.P.M. & GUNN, J.M. (1980). The Tamoxifen Trial-A double-blind comparison with stilboestrol in postmenopausal women with advanced breast cancer. *Eur. J. Cancer*, **1**, 83 (Suppl).

TAYLOR, R.E., POWLES, T.J., HUMPHREYS, J. & 5 others (1982). Effects of endocrine therapy on steroid-receptor content of breast cancer. *Br. J. Cancer*, **45**, 80.

WARD, H.W.C. (1973). Anti-oestrogen therapy for breast cancer: A trial of tamoxifen at two dose levels. *Br. Med. J.*, **1**, 13.

WATERFIELD, M.D., SCRACE, G.T. & WHITTLE, N. (1982). A monoclonal antibody to the human epidermal growth factor receptor. *J. Cell. Biochem.*, **20**, 140.